# Perceptions of adolescents’ sexual and reproductive health and rights: a cross-sectional study in Lahore District, Pakistan

**DOI:** 10.1186/s12914-017-0113-7

**Published:** 2017-02-23

**Authors:** Sarosh Iqbal, Rubeena Zakar, Muhammad Zakria Zakar, Florian Fischer

**Affiliations:** 1 Institute of Social and Cultural Studies, University of the Punjab, P.O. Box 54590, Lahore, Pakistan; 2 Department of Public Health Medicine, Bielefeld University, School of Public Health, P.O. Box 100 131, 33501 Bielefeld, Germany

**Keywords:** Adolescents, Sexual and reproductive health, Rights, Perception, SRHR

## Abstract

**Background:**

Sexual and reproductive health (SRH) is a significant aspect of adolescents’ growth, safeguarded by SRH rights (SRHR). Despite various global efforts to promote adolescents SRHR (ASRHR), the majority of adolescents still lack awareness and autonomy to access SRH related information and services. This research aimed to explore the knowledge and perceptions of adolescents’ sexual and reproductive health rights and highlights key constraints hindering adolescents from accessing and exercising SRHR in the district of Lahore, Pakistan.

**Methods:**

The research uses a mixed methods approach including both quantitative and qualitative methods. For quantitative component, household survey was conducted with 600 respondents including adolescents (15–19 years) and their parents/caregivers. A multistage cluster random sampling technique was performed, based on the population proportion of administrative towns in Lahore district, Pakistan. A structured interview schedule was used to collect data. Quantitative data were collected by a standardized quantitative questionnaire; analysis was performed using SPSS version 21. For qualitative data collection, 12 in-depth interviews with teachers and doctors and four focus group discussions with adolescents were conducted, and analysed using thematic areas.

**Results:**

The research revealed a low level of perception of ASRHR amongst the respondents and identified socio-cultural and structural constraints as the major underlying issues. Although more than half of the respondents were found to be aware of ASRHR, agreed to their importance and were in favour for adolescents to have access to requisite information, nonetheless they believed that adolescents had limited ability to exercise these rights.

**Conclusions:**

The research found a low level of perception amongst adolescents and their parents/caregivers about ASRHR in Lahore district emphasising the rights-based approach. There is an urgent need to design specific policies and educational programmes to promote healthy practices. Research is recommended to inform and advocate Punjab Government and communities, including partners, teachers, doctors, religious scholars and media groups, to empower adolescents through health education. This can be achieved through the inclusion of SRH topics in educational curricula, establishing a virtual knowledge centre, encouraging debate competitions, and organising orientation sessions for professionals/experts and community etc.

## Background

Sexual and reproductive health (SRH) is a significant aspect of adolescents’ growth, and it is safeguarded by sexual and reproductive health rights (SRHR), promoting equality and dignity. These were defined under the ‘rights-based approach’ at the 1994 International Conference on Population and Development (ICPD) and are also recognised in a similar way to human rights in the 96th article of the 1995 Beijing Platform for Action [1–3]. Adolescent SRHR are often confused with SRH; nevertheless, they are different, as stated by the ICPD. SRH refers to complete physical, mental and social well-being in all aspects related to sexual and reproductive growth, whereas SRHR covers a range of rights to autonomy and to access SRH information and services [4]. In 1994, the International Planned Parenthood Federation Charter outlined key ﻿rights of adolescent SRHR (ASRHR), including the rights to healthcare, information and education, life, liberty, privacy, freedom of thought, equality, choice in marriage and number of children, freedom from torture and access to the benefits of scientific research [5].

Since the ICPD, the global community has made key strides in promoting ASRHR; however, millions of disadvantaged adolescents lack the autonomy to access comprehensive SRH information and services, due to the taboo nature of the subject [6, 7]. Adolescence, defined as the period between 10 and 19 years of age, is associated with a multitude of dramatic changes, leading to curiosity and experimentation, during which adolescents are not well informed about their SRH needs [8, 9]. Moreover, the taboo status of SRHR and the lack of a conducive environment, particularly for acquiring practical knowledge and services to protect themselves from negative outcomes, make adolescents more vulnerable than adults to infections, exploitation and abuse [10].

In the developing country of Pakistan, adolescents make up 23% of the population [11]. Nevertheless, they have poor access to SRH knowledge and services, which is evident from the trends of early marriage, unintended pregnancy, gender discrimination, violence, and low rates of contraception and literacy [10, 12]. Within the Islamic context of Pakistan, the subject of human sexuality is considered a societal taboo and associated with strong ideology and moral values, restricting open discussion [13]. Also, there are various misconceptions, especially among lower and middle-income groups that unmarried and under 18 years of adolescents are too young to require SRH-related information and services [14]. Furthermore, laws and policies are generally restrictive and environment is not conducive to acknowledge ASRHR for healthy development [10, 15].

The available literature suggests that adolescents have a limited understanding of SRH, and in particular about SRHR in Pakistan [10, 11]. Although few studies examined the adolescents knowledge regarding content of SRH rights and autonomy [16, 17], nonetheless, the perception of adolescents’ sexual and reproductive health rights is under-researched in Pakistan, primarily due to the lack of access to relevant information, education, services and support mechanisms [14, 18]. Given this backdrop, this research was designed to explore the knowledge and perception of adolescents’ sexual and reproductive health rights and highlights the key constraints hindering adolescents from accessing and exercising SRHR in the district of Lahore.

## Methods

### Research setting and design

The research was conducted in the district of Lahore, the provincial capital of Punjab Province (Pakistan). The total population of the district is 16,776,000 with a large number of adolescents. Administratively, the district is divided into nine towns, consisting of 274 union councils [19].

This was a cross-sectional study which applied a mixed-methods explanatory design, wherein both quantitative and qualitative data collection were conducted concurrently to generate a more holistic understanding to aid in drawing conclusions [20]. Research adopted the complementarity rationale for mixed method approach to examine interconnected and distinct aspects of the phenomenon [21]. We carried out a household survey with late adolescent boys and girls (aged 15–19 years) and their parents/caregivers. In-depth interviews (IDIs) were conducted with male and female teachers and doctors/lady doctors, and focus group discussions (FGDs) were carried out with adolescents, for helping to explain the quantitative findings.

Considering the nature of the topic and local cultural values, the late adolescence age group (15–19 years) and three key stakeholders (parents, teachers and doctors) were included in the research. The rationale for selecting the latter lay in the fact that, taken together, these groups contribute to create an enabling environment for providing SRH information and services to adolescents. For instance, parents/caregivers are the primary source of information at family level and they influence adolescents’ growth. Likewise, academic teachers are influential through health education at school/college level while doctors provide SRH services at health facility level [22].

### Sample size

A sample size of 300 households was determined for the survey, using the Cochran formula $n=\frac{z_{a/2}^2\cdot p\cdot \left(1- p\right)}{d^2}$ assuming a 50% anticipated population proportion of adolescents (*p*) to obtain the most conservative sample size estimate [23]. Along with this, 95% level of significance (*z*), 95% level of confidence (*α*), 10% absolute precision required on either side of the proportion (*d*), and design effect of 3.0 was accounted for sampling. This formula yielded sample size of 288, with 9.6 households per cluster, which was rounded off to 10 households per cluster, giving total sample size of 300. The target sample size with 300 households was chosen by considering the minimal number necessary to respect the Central Limit Theorem, a key principle in statistics for cost-effectiveness. This is also similar to the EPI 30-cluster sample method developed by the World Health Organisation and UNICEF for population-based surveys [24]. Given the nature of the subject, we added a 5% non-response rate. Hence, the sample size was set at 315 households before going into the field.

We adopted a multistage cluster sampling technique. As a first step, a union council wise list of all urban blocks/wards and villages/*mouzas*/*dehs* was obtained from the administrative towns. These blocks and villages were taken as geographical clusters and selected using a random number list from the statistical package Epi-Info, version 6.0. Subsequently, 10 eligible households that included adolescents aged 15–19 years were randomly selected and interviewed within each cluster. The parents/caregivers of adolescents living in the same household were also interviewed, thus achieving the required total of 300 households, with 600 respondents, including 300 adolescents and 300 parents/caregivers.

In order to select the first household, the field teams used a random number table or rotated a pencil to establish the direction from the central location of the cluster, e.g. the largest building. The direction of the sharpened end of pencil or last digit of the random number table indicated the first household. Then, the data collection was started from the 4th household, being central to a quadrant of cluster to decrease cluster homogeneity through randomisation [20]. Then we moved to the nearest household and this was repeated until the required number of respondents had been interviewed in the cluster. In case, where one household had more than one eligible adolescent, the elder one through oldest-adult method was selected for interview [25].

For the qualitative research, purposive sampling was adopted for selection and recruitment of the information-rich participants, who were well acquainted about particular phenomenon of interest, nevertheless, possess similar social cultural background [26]. The purposive sampling was drawn with the help of local outreach healthcare providers and community gatekeepers [27], who helped the researchers in identifying the potential participants. These participants were invited to participate in research and interviews/discussions were scheduled with interested participants after seeking consent. Participants for FGDs were recruited based on their similar age and gender to create relatively homogenous groups. FGDs with adolescents were held in community settings while IDIs with teachers and doctors were held in their workplace.

A total of 12 IDIs were conducted with academic and medical professionals, including 6 teachers and 6 doctors (having three males and three females in each group), to seek detailed insight into the topic. This small number of IDIs was conducted to complement quantitative findings [28]. The selection criteria for participants included male and female teachers at schools/colleges teaching the late adolescence age group and general physicians/doctors/lady doctors serving in the public or private sector. In addition, four FGDs were carried out with 12 boys and 12 girls aged 15–19 years to collect intensive information through creating a friendly environment [29].

### Data collection tool and procedures

After consulting previously implemented assessment tools on ASRHR in Pakistan and other geographical settings, we adopted a structured interview schedule and semi-structured guides for IDIs and FGDs [10, 30–32]. These contained questions on awareness of the content of ASRHR, degree of importance given to knowing about ASRHR, accessibility to sources, autonomy to exercise rights, underlying constraints faced by adolescents and strategies to overcome them. Questions regarding the contribution of teachers and doctors when approached by adolescents were added to the qualitative guides. The interview schedules and guides were further translated into the local language, Urdu, in order to conduct the face-to-face cross-sectional survey with adolescents and parents/caregivers, the IDIs with teachers and doctors, and FGDs with adolescents.

The data was collected during January and February 2014 in the district of Lahore (Pakistan), utilising four field teams, each of which consisted of both male and female interviewers, accompanied by researchers. Qualified interviewers (a minimum of intermediate level) with similar experience were selected. The field teams received 2 days of training before data collection on the content of questionnaires/guides, the selection of eligible respondents/participants, data accuracy, completeness and ethical requirements. Researchers closely monitored and supervised the field teams during their fieldwork through surprise visits. The responses were noted on the questionnaires while the proceedings were audio recorded and supplemented with note-taking during IDIs and FGDs.

Overall, we achieved a high response rate. About 4% of the survey respondents refused to participate and 2% skipped questions giving only a few responses for analytical purpose, and were, therefore, excluded from the research. Thus, additional households were approached for replacement, leading to overall 600 respondents. Regarding qualitative research, it was necessary to approach 30 adolescents and seven female teachers in order to complete the sample of 24 adolescents and three female teachers.

### Measurement of variables

#### Outcome variable

The outcome variable was the perception of ASRHR. Objectively, perception is difficult to measure [33]. It involves the process of selection, organisation and interpretation of information through awareness of cues and behavioural intentions that occur in social situations. There have been various previous studies that examined different aspects of social perception [34, 35]. However, our research focused on the combination of awareness, the importance they attributed to knowing about ASRHR (illustrating intention) and access to sources of information (environment), along with the ability to exercise (autonomy), which is an important indicator for the implementation of rights [36]. Hence, the level of perception was measured for this research through computing four variables: awareness about ASRHR, degree of importance, accessibility and autonomy, as described below.

A series of closed questions about each of the ASRHR was asked to determine awareness, e.g. ‘do you think that the right to healthcare is one of the ASRHR?’ Possible responses were 'yes', 'no', and 'don’t know'. Later on, all 11 ASRHR were computed to develop an awareness index to measure the overall awareness level of ASRHR through the median [37]. Scores ranged from 0 to 11 and gave a median value of 9 for both adolescents and parents/caregivers. Thus, a respondent’s score equivalent to the median or above was considered a high level and below the median a low level of awareness.

All the respondents were asked the following question: ‘Do you think it is important for adolescents to know about these SRHR?’ Responses were grouped into yes/no, followed by the question: ‘To what extent do you agree or disagree that such knowledge is important?’, wherein a 5-point Likert scale was used, i.e. strongly agree, agree, neither agree nor disagree, disagree and strongly disagree.

In order to determine adolescents’ access to the relevant information, respondents were initially asked: ‘In your opinion, do adolescents have access to relevant sources of information about SRHR or not?’. The given responses included yes/no. If the answer was ‘yes’, multiple responses were collected for the question: ‘From where do adolescents get information about their SRHR?’ Responses included: parents/caregivers, teachers, friends, media/internet, siblings/cousins, religious leaders and service/healthcare providers. However, if the original answer was 'no', the interviewer skipped to another question describing constraints.

To measure the adolescents’ ability to exercise their SRHR, respondents were asked, ‘In your opinion, are adolescents free to exercise their SRHR or not?’, where the given responses included 'yes'/'no'.

A question related to constraints was asked twice, when respondents had answered ‘no access’ and ‘no autonomy’ as mentioned above. This question was: ‘Which main difficulties do adolescents face?’ It included multiple responses, further grouped into three categories: socio-cultural constraints (religious taboos/limitations, prohibited topic of discussion, traditional beliefs and practices), economic constraints (non-affordability of SRH-related services) and structural constraints (lack of appropriate literature or formal SRH education within the curriculum and non-availability of SRH services).

Lastly, respondents were asked: ‘Whom do adolescents consult to overcome these constraints?’ Responses included: parents/caretakers, siblings/cousins, friends and teachers.

Hence, the outcome variable of ‘adolescent and parental level of perception’ was developed after computing four binary variables, i.e. awareness, degree of importance, accessibility and autonomy with yes/no response options. Respondents scoring 3–4 (considering the median value) were labelled as having a high level of perception, whereas a score of 0–2 was taken as a low level of perception.

### Explanatory variables

The explanatory variables consisted of socio-demographic status, comprising: adolescent and parental age (in years), educational status of adolescents (primary/middle/matric/intermediate/ graduation or above) and parents (educated/uneducated), earning member of family (yes/no), parental occupation (unemployed/government/private employee/business/farming/labourer/daily waged/retired), family structure (nuclear/joint), number of children in family (1–2/3–4/5 or above) and household wealth status (poor, middle and rich). Wealth status was constructed using household assets and given weight by the price-of-item approach or on an ad-hoc basis [38]. These include: type of housing, floor materials used, wall materials used, toilet facilities, source of drinking water, household necessities such as radio, television, mobile or fixed phones, mode of transportation (car/bicycle/motorcycle/donkeys), communications and monthly income etc. All scores were accumulated to generate household wealth tertile and categorized as poor (lowest third), middle (medium) and rich (highest third) households [39, 40].

### Data processing and analysis

Quantitative data was coded and entered into EpiData software to ensure data quality through applying legal values, range checks and rules of validation. Data was cleaned for consistency and afterwards exported to SPSS, version 21, for analysis. The clustered nature of data was ignored considering the simplest approach for analysis [41]. Frequency distributions and summary statistics were estimated using design-based analysis, in the form of percentages and cross-tabulations. In the univariate analysis, socio-demographic characteristics and the perception variables were expressed in percentages. However, in the bivariate analysis, cross-tabulations were used to identify the distribution of outcome variables among the selected characteristics, while the chi-square test of association was applied to measure the statistical significance.

Qualitative data was initially transcribed and coded, followed by checking and validating the audio tapes to ensure accuracy. A thematic approach was adopted for analysis. The process combined an inductive and deductive approach. Therefore the thematic approach was driven in part by the data and in part by a pre-existing framework based on a preliminary list of codes which was developed and refined during the research process. Major themes and sub themes that emerged during the process of data collection were also identified and summarised, nevertheless the relevant themes to our area of interest were used, focusing on knowledge and perception of ASRHR and underlying constraints.

### Ethical considerations

Ethical approval for this research was obtained from the Institutional Review Board, University of the Punjab, Lahore (Pakistan). Each adult respondent/participant signed an informed consent, indicating their willingness to participate in the research, while the consent of adolescents less than 18 years of age was obtained from their respective parent/caregiver only [42]. Respondents/participants were also informed of their right to refuse to participate or to withdraw at any time. Moreover, the privacy and confidentiality of their responses was also assured by coding each participant with a unique ID.

## Results

### Socio-demographic characteristics of respondents/participants

Adolescents and their parents/caregivers from 300 households in the district of Lahore took part in the study. Table 1 presents the socio-demographic characteristics of respondents. The mean age of adolescents was found to be 17 years and that of parents/caregivers was 47 years. Approximately 33% of adolescents had completed their secondary level education and 27% had completed intermediate level. Meanwhile, 75% of parents/caregivers were found to be educated. Regarding employment status, the majority of parents/caregivers (66%) were employed, while a small number of adolescents (11%) were employed. More than half of the households (55%) lived in joint family systems; 44% of adolescents had 3–4 siblings. Most of the respondents (67%) had middle wealth status, while 22 and 11% belonged to the rich and poor status, respectively.

**Table 1:** Socio-demographic characteristics of surveyed households (*n* = 300)

Characteristics	n	(%)
Adolescents age		
Median (range)	18 (15–19)	
M (SD)	17.31 (±1.33)	
Education status		
Uneducated	20	6.7
Primary/Madrassa	17	5.7
Middle	45	15.0
Secondary/Matric	98	32.7
Intermediate	81	27.0
Graduate	39	13.0
Adolescents as earning member of family		
Yes	32	10.7
No	268	89.3
Family structure		
Joint family	166	55.3
Nuclear family	134	44.7
Number of siblings		
Median (range)	2 (1–9)	
M (SD)	2.17 (±0.73)	
1–2	58	19.3
3–4	132	44.0
5 and above	110	36.7
Parental age		
Median (range)	48 (30–70)	
M (SD)	46.96 (±6.78)	
Parental education		
Uneducated	74	24.7
Educated	226	75.3
Parental occupation		
Unemployed	102	34.0
Government/Private employee	82	27.3
Business/Farming	61	20.3
Labourer/Daily wager	50	16.7
Retired	5	1.7
Household wealth status		
Rich	67	22.5
Middle	199	66.8
Poor	32	10.7

The qualitative research participants included six doctors/lady doctors (aged 38–55 years with 12–25 years of clinical practice experience), six male/female teachers (aged 27–54 years with 4–20 years of teaching experience with adolescents) and 24 adolescents boys/girls aged 15–19 years having a mixed background of educated and uneducated from school/colleges and neighbourhood (*mohallah)* settings.

### Level of awareness

**Fig. 1:**
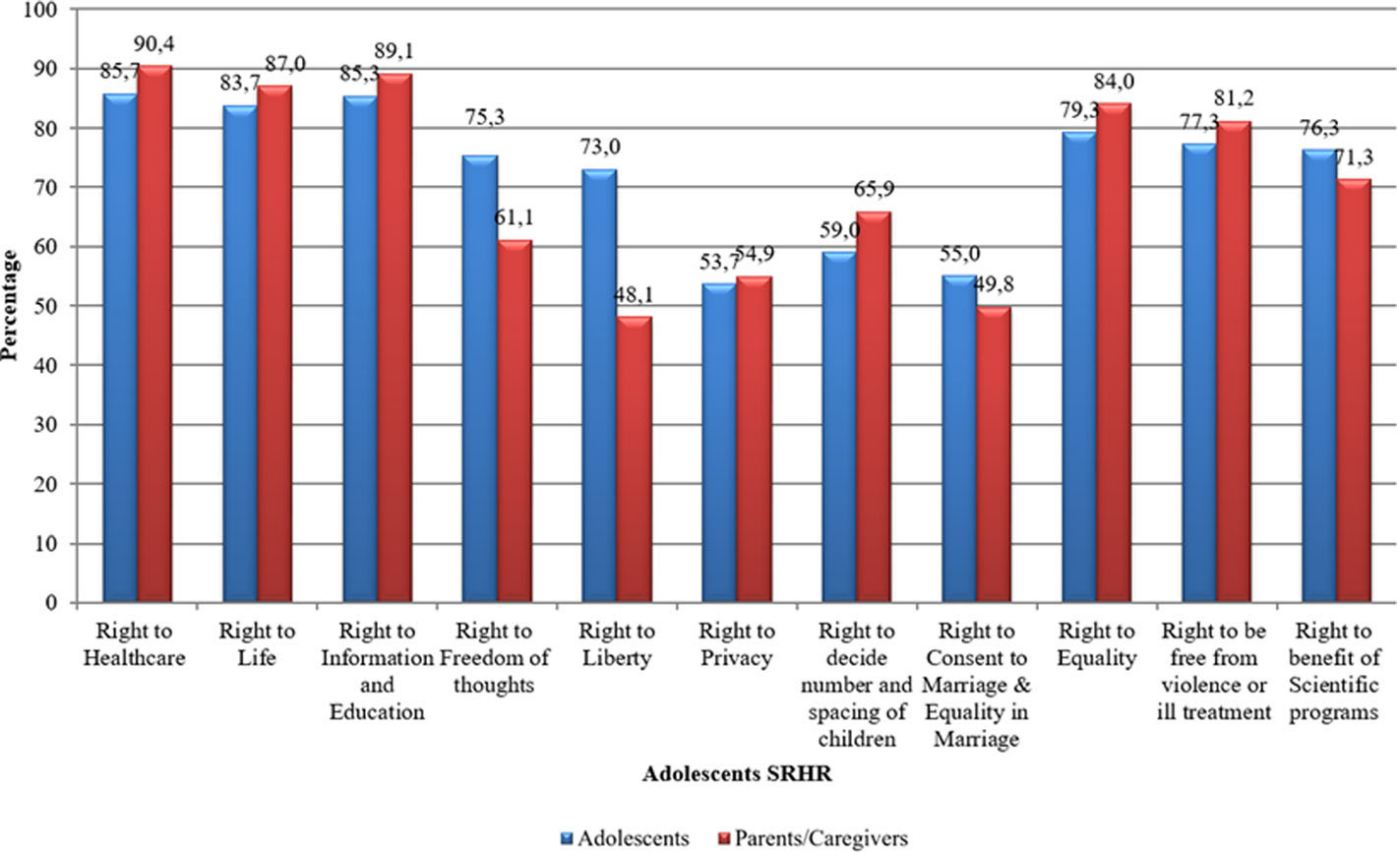
Respondents awareness about each of adolescents sexual and reproductive health and rights

Figure 1 llustrates the respondents’ awareness of each of the 11 ASRHR. The most widely known rights were found to be the rights to healthcare, education, life and equality. Nevertheless, opinions of respondents regarding rights to liberty and freedom of thoughts were slightly contradictory.

Based upon the awareness index, developed after computing responses on all ASRHR to determine the overall awareness with binary category of low/high level based on median value as cut-off point, the results show that more than half of the respondents (52% of adolescents and 51% of parents/caregivers) were found aware of ASRHR.

Contrarily, the qualitative findings from the IDIs and FGDs revealed low awareness amongst teachers and adolescents, while doctors’ awareness was found to be comparatively high. Most of the doctors, nonetheless only few teachers and adolescents could list some of the contents of 11 ASRHR. Overall, a poor knowledge of the content of ASRHR was found in adolescents, as expressed by one 18-year-old girl:

*It’s difficult to list all the ASRHR. We don’t know about these rights, and nobody has told us.*

Regarding their contribution to enhancing adolescents’ awareness about ASRHR, the doctors affirmed that often girls and boys approach them quietly and hesitantly to seek advice on their SRH issues, particularly for pubertal and hormonal changes. Most of the teachers stated that they avoid such discussions due to shyness and reluctance.

### Degree of importance

**Fig. 2:**
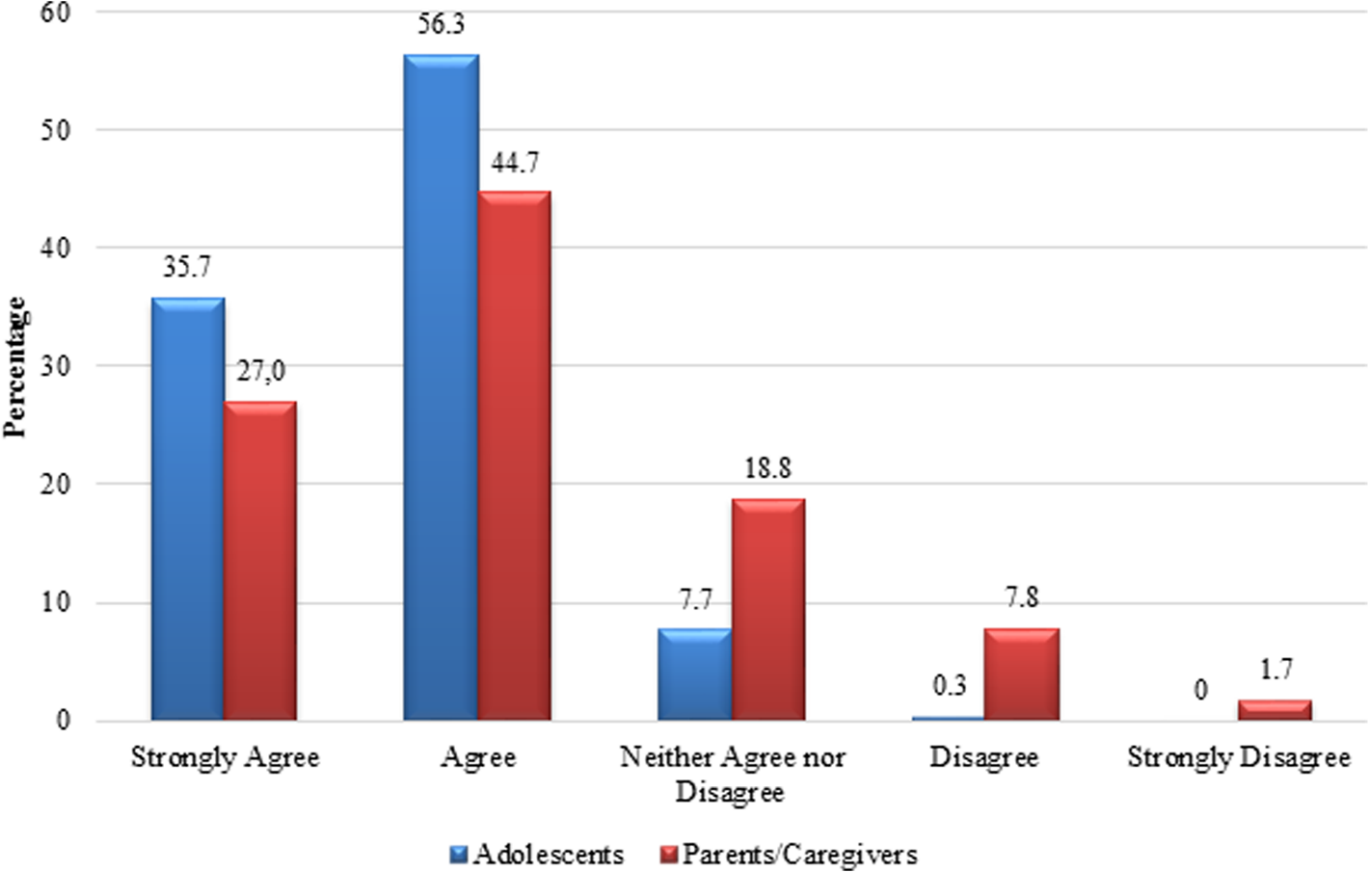
Respondents views on importance for adolescents to know about ASRHR

A majority of adolescents (92%) and parents/caregivers (70%) reported that adolescents should know about their SRHR beforehand. Moreover, 56% of adolescents and 45% of parents/caregivers agreed, while 36% of adolescents and 27% of parents/caregivers strongly agreed to the importance of ASRHR, as depicted in Fig. 2.

Similarly, the qualitative research showed that almost all participants including doctors, teachers and adolescents were in favour, emphasising that these rights cannot be ignored, especially in the current era of information technology. A 53-year-old doctor, having more than 25 years of clinical experience and practising in a public-sector hospital stated:

*Yes, of course, these rights are most important for the adolescent age group, particularly to maintain an improved health status, enabling them to act confidently. I strongly encourage adolescents to have an awareness of their SRHR, such as the rights to education, health and consent to marriage.*

A 19-year-old girl studying for a BA (part 1) stated:

*These rights are very important as they enable us to know which types of rights we have, being in this age group and how to protect ourselves and live a good life through exercising these rights.*

### Access to sources of information

Table 2 shows that approximately 56% of adolescents and 53% of parents/caregivers confirmed that adolescents have access to SRH-related information. Among people having access, 71% of adolescents and 64% of parents/caregivers reported friends as the most common sources of information, followed by parents/caregivers, teachers, siblings/cousins and the media.

**Table 2:** Sources of information to access information and main constraints and overcoming strategies regarding perception of ASRHR (*n* = 300)

Characteristics	Adolescents (*n* = 300)	Parents/Caregivers (*n* = 300)
	(in %)	(in %)
Access to sources of information	55.7	53.4
Sources of information^a^		
Parents/caregivers	47.7	59.7
Siblings/cousins	29.0	46.0
Friends	71.3	64.3
Teachers	33.7	31.0
Religious leaders	13.3	17.3
Service/healthcare providers	9.3	16.0
Media/Internet	17.7	33.7
Ability to exercise ASRHR	38.3	39.7
Main constraints identified		
Socio-cultural constraints	67.0	80.7
Economical constraints	4.3	59.0
Structural constraints	52.0	74.0
Overcome strategies		
Consult with parents/caregivers	58.3	45.7
Consult with siblings/cousins	43.3	33.7
Consult with friends	72.3	48.0
Consult with teachers	23.7	14.0

^a^Percentages refer to those responding having access to information

Likewise, most of the participants of IDIs and FGDs also acknowledged that adolescents had access to SRHR information. All doctors and teachers identified friends primarily and the media/internet as secondary sources to obtain the requisite information. Nevertheless, they were concerned about the authenticity and accuracy of information acquired through friends and the internet, as explained by a 35-year-old female teacher at a private school:

*Adolescents get information from their surroundings, particularly from friends, the media, and the internet, but here the reliability of information is doubtful, particularly from friends as they belong to the same age group and are experiencing the same things. Thus, it increases the risk of exploitation, harm and misinformation.*

All the doctors also highlighted that adolescents usually come to them for medical advice on common SRH issues, such as the menstrual cycle, hygiene, pubic and facial hair etc. Moreover, some of the teachers also stated that girls approach them.

Similarly during the FGDs, some of the adolescents also affirmed that they often access the internet or ask friends and elder siblings/cousins to acquire such information. Girls mentioned mothers as their main source of information; however, here some contradictory opinions were also seen, as one 18-year-old girl studying at college explained:

*We usually access our friends, elder sisters/cousins and the internet. The internet has answers to our all questions. Our parents, especially our mothers, don’t discuss these issues with us, rather they remain silent, shy and unfriendly on such occasions.*

### Ability to exercise SRHR

The results shown in Table 2 also reveal that the more than half of the adolescents (62%) and parents/caregivers (60%) reported about adolescents’ inability to freely exercise their SRHR. These are similar to the qualitative findings, wherein most of the participants spoke about the limited autonomy of adolescents. A boy, having a *madrasah* education, said:

*How can we exercise these rights when we have no awareness or knowledge about our rights?*

One 45-year-old doctor, who has been practising for the last 15 years, had a different opinion and remarked:

*Having limited knowledge, adolescents are unable to exercise their rights within our society. However, this situation is different in urban areas where adolescents are more literate, educated and knowledgeable, having more potential to exercise them than in rural areas.*

All doctors and teachers also emphasised supporting adolescents in exercising their ASRHR, such as the right to higher studies, healthcare, consent in marriage and gender equality, through counselling and dialogues with parents/caregivers, where needed.

### Perceptions of ASRHR

The results showed a low level of perception amongst more than half of the parents/caregivers (58%) and adolescents (55%). Similarly, the qualitative findings also highlighted a low perception of ASRHR amongst all adolescents, teachers and few doctors. One 54-year-old doctor with 20 years’ experience in a private-sector hospital said:

*We all have a low perception as our families and teaching institutes are not well-equipped, having deficient knowledge about ASRHR. Although we know about a few rights, we are ignorant of most of them, so we keep adolescents deprived of all those rights which they are entitled to have at their increasing age in order to become a confident adult.*

A girl of 18 years with 12 years of schooling also shared her feelings:

*How can our perception of SRHR be shaped when our parents and teachers remain silent on these issues? Whenever we are in a fix about some SRH-related problem, like the menstrual cycle, sexual abuse or even choice of education and consent to marriage, we face difficulties and embarrassment about where to go and whom to consult. Our quest about these issues gives us a guilty conscience and makes us confused, forcing us to approach unreliable sources and adopt unhealthy practices.*

### Constraints and strategies

Table 2 also revealed the underlying constraints faced by adolescents in accessing or exercising their ASRHR. Overall, 81% of parents/caregivers and 67% of adolescents identified socio-cultural constraints. Additionally, 74% of parents/caregivers and 52% of adolescents reported structural constraints, while 59% of parents/caregivers but only 4% of adolescents identified economic constraints.

The qualitative findings also highlighted the various socio-cultural constraints hindering adolescents from accessing or exercising ASRHR. These included societal and cultural inhibitions related to the sensitivity of the topic, taboos on discussion, shyness/hesitation and traditional practices. Most of the doctors emphasised the teachings of Islam as a complete code of conduct providing guidance in all matters of life and taking insight from the teachings of the Koran. Moreover, a male teacher aged 27 years teaching students aged 10–16 years further added:

*Adolescents’ perceptions of SRHR are subject to the disparities of social class, negative trends in society and their environment. Adolescents belonging to the elite class are more aware, whereas the middle class has to fight for these rights and the lower class is not privileged at all.*

Regarding the various structural constraints, participants identified the lack of a conducive environment, no appropriate/authentic literature and a communication gap between adolescents and their parents. They also highlighted the poor performance of government, particularly in the health and education sector for failing to promote SRH as a universal subject. Participants also acknowledged financial constraints as a barrier to health advice for adolescents. A 38-year-old lady doctor practising for the last 10 years explained:

*The high fees charged by professionals/experts limit adolescents’ capacity for consultation. This increases their preference to approach quacks and pay them much high fee but in sequels and also become victim of various problems, without having adequate or authentic knowledge about the issues.*

In order to deal with these constraints, 72% of adolescents and 48% of parents mentioned that adolescents mostly consult with their friends. Furthermore, 58% of adolescents and 46% of parents/caregivers opined that adolescents also consult with their parents/caregivers. The qualitative findings also favoured consulting with friends or siblings to cope with constraints, while some girls also mentioned consulting their mothers for support.

### Relationship between socio-demographic characteristics and level of perception of ASRHR

Tables 3 and 4 present the bivariate analysis of socio-demographic variables against adolescent and parental perception about ASRHR and its related variables (awareness level, degree of importance, accessibility, autonomy). The findings of both tables showed a high level of perception in adolescents of 15 and 18 years of age, having educated parents, living in the joint family system, with 5 and above siblings and belonging to the rich wealth status. The same pattern was seen in the case of awareness, degree of importance, accessibility and autonomy. The background characteristics of parental educational status and wealth status were all found to be statistically significant (*p* ≤ 0.05), with some of the variables shaping perceptions of ASRHR, nevertheless adolescents age and education status was not found significant with perception of ASRHR.

**Table 3:** Relationship between socio-demographic status and adolescents’ perception about ASRHR (*N* = 300)

Characteristics		Awareness		Importance		Access to information		Free to exercise		Perception		
	Total	High	*p*-value*	Yes	*p*-value*	Yes	*p*-value*	Yes	*p*-value*	High	Low	*p*-value*
	n (%)	(in %)		(in %)		(in %)		(in %)		(in %)	(in %)	
Adolescents’ age												
15 years	39 (13.0)	28.2	<0.01	89.7	0.34	66.7	0.05	56.4	0.09	53.8	46.2	0.31
16 years	54 (18.0)	51.9		94.4		57.4		40.7		46.3	53.7	
17 years	42 (14.0)	35.7		90.5		61.9		40.5		40.5	59.5	
18 years	104 (34.7)	58.7		95.2		57.7		31.7		48.1	51.9	
19 years	61 (20.3)	65.6		86.9		39.3		34.4		34.4	65.6	
Adolescents’ education status												
Uneducated	20 (6.7)	50.0	0.08	100	0.23	55.0	0.91	45.0	<0.01	60.0	40.0	0.26
Primary/Madrassa	17 (5.7)	47.1		100		58.8		52.9		52.9	47.1	
Middle	45 (15.0)	33.3		88.9		55.6		57.8		51.1	48.9	
Secondary/Matric	98 (32.7)	53.1		92.9		57.1		40.8		44.9	55.1	
Intermediate	81 (27.0)	61.7		92.6		50.6		23.5		42.0	58.0	
Graduate	39 (13.0)	51.3		84.6		61.5		30.8		30.8	69.2	
Earning member of family												
Yes	32 (10.7)	50.0	0.84	100	0.07	50.0	0.49	56.3	<0.02	56.3	43.8	0.16
No	268 (89.3)	51.9		91.0		56.3		36.2		43.3	56.7	
Parental education												
Uneducated	74 (2 4.7)	39.2	<0.01	79.7	<0.01	47.3	0.09	37.8	0.92	31.1	68.9	<0.01
Educated	226 (75.3)	55.8		96.0		58.4		38.5		49.1	50.9	
Family structure												
Joint family	166 (55.3)	51.2	0.85	89.8	0.11	56.6	0.71	42.8	0.07	47.6	52.4	0.25
Nuclear family	134 (44.7)	52.2		94.8		54.5		32.8		41.0	59.0	
Number of siblings												
1–2	58 (19.3)	34.5	<0.01	91.4	0.94	51.7	0.79	37.9	0.94	37.9	62.1	0.49
3–4	132 (44.0)	49.2		91.7		56.8		39.4		45.5	54.5	
5 and above	110 (36.7)	63.6		92.7		56.4		37.3		47.3	52.7	
Household wealth status												
Rich	67 (22.5)	71.6	<0.01	92.5	0.94	53.7	0.71	37.3	0.38	58.2	41.8	<0.04
Middle	199 (66.8)	46.2		92.0		55.8		40.7		41.7	58.3	
Poor	32 (10.7)	46.9		90.6		62.5		28.1		37.5	62.5	

*Chi-square test was applied to measure *p*-value

**Table 4:** Relationship between socio-demographic status and parents/caregivers’ perception about ASRHR (*N* = 300)

Characteristics		Awareness		Importance		Access to information		Free to exercise		Perception		
	Total	High	*p*-value*	Yes	*p*-value*	Yes	*p*-value*	Yes	*p*-value*	High	Low	*p*-value*
	n (%)	(in %)		(in %)		(in %)		(in %)		(in %)	(in %)	
Adolescents’ age												
15 years	39 (13.0)	43.6	0.21	71.8	0.13	46.2	0.84	53.8	0.17	46.2	53.8	0.42
16 years	54 (18.0)	63.0		70.4		57.4		31.5		44.4	55.6	
17 years	42 (14.0)	50.0		59.5		52.4		45.2		38.1	61.9	
18 years	104 (34.7)	52.9		77.9		52.9		35.6		47.1	52.9	
19 years	61 (20.3)	42.6		62.3		49.2		36.1		32.8	67.2	
Adolescents’ education status												
Uneducated	20 (6.7)	60.0	0.24	90.0	0.14	50.0	0.93	45.0	<0.01	55.0	45.0	0.42
Primary/Madrassa	17 (5.7)	47.1		82.4		52.9		41.2		41.2	58.8	
Middle	45 (15.0)	37.8		64.4		53.3		60.0		51.1	48.9	
Secondary/Matric	98 (32.7)	58.2		73.5		49.0		35.7		41.8	58.2	
Intermediate	81 (27.0)	51.9		64.2		56.8		28.4		40.7	59.3	
Graduate	39 (13.0)	43.6		64.1		48.7		38.5		30.8	69.2	
Earning member of family												
Yes	32 (10.7)	56.3	0.53	87.5	<0.01	53.1	0.89	50.0	0.16	56.3	43.8	0.09
No	268 (89.3)	50.4		67.9		51.9		37.3		40.7	59.3	
Parental education												
Uneducated	74 (2 4.7)	47.3	0.46	59.5	<0.01	39.2	<0.01	27.0	<0.01	29.7	70.3	<0.01
Educated	226 (75.3)	52.2		73.5		56.2		42.5		46.5	53.5	
Family structure												
Joint family	166 (55.3)	50.0	0.70	68.1	0.41	53.0	0.69	42.8	0.10	44.6	55.4	0.38
Nuclear family	134 (44.7)	52.2		72.4		50.7		33.6		39.6	60.4	
Number of siblings												
1–2	58 (19.3)	44.8	0.45	65.5	0.35	48.3	0.06	31.0	0.38	34.5	65.5	0.38
3–4	132 (44.0)	54.5		74.2		46.2		39.4		43.2	56.8	
5 and above	110 (36.7)	50.0		67.3		60.9		41.8		45.5	54.5	
Household wealth status												
Rich	67 (22.5)	62.7	0.09	68.7	0.17	61.2	0.13	37.3	0.78	58.2	41.8	<0.01
Middle	199 (66.8)	48.7		72.4		48.2		40.2		38.7	61.3	
Poor	32 (10.7)	43.8		56.3		59.4		34.4		34.4	65.6	

*Chi-square test was applied to measure *p*-value

## Discussion

This research aimed to explore the knowledge and perception of adolescents’ sexual and reproductive health rights and highlights the key constraints hindering adolescents from accessing and exercising SRHR in the district of Lahore (Pakistan). This research is unique in nature as it focused on knowledge and perceptions of basic ASRHR within the community of Pakistan, where discussion on such topics is prohibited.

Adolescents have the right to acquire SRH information and services. Nonetheless, in a developing country like Pakistan, where these rights are infringed, a lack of perception about rights could lead to sexual harassment, violence, life-long psychological damage and/or negative health outcomes [18]. The findings of this research broadly revealed that there is a dearth of ASRHR perception amongst adolescents and their parents in the district of Lahore due to various contributing factors. Particularly, it highlighted a difference in qualitative and quantitative findings regarding overall perception and the level of awareness of SRHR content, wherein respondents claimed to know about ASRHR as per quantitative results. However, when interviewed and probed in depth and when the subject was discussed in groups, respondents actually had limited awareness and perception, which is similar to the results of studies conducted previously in Pakistan and Nigeria [32, 43, 44]. An ambiguous understanding was even seen in educated parents, who have the foundational responsibility to guide adolescents. This finding is similar to those of a study conducted in Eastern Europe and the Central Asia region [45].

This research found that respondents also stressed the importance of knowing about ASRHR, even claiming it to be indispensable. It was found that half of the adolescents had access to the requisite information, but practising their rights was very rare [46]. This indicated the acceptability for adolescents to know about SRHR, regardless of the sensitivity and taboos associated with the topic, as exemplified in the SRHR assessment framework of the World Population Foundation, Pakistan [16].

Regarding the various sources of ASRHR information, the findings of this research were not very different from those of other research conducted in Pakistan and similar countries like Bangladesh, where friends and the media/internet emerged as the most common sources of information. However, the quality of information passed on through friends, who are of the same age group, and the internet remains questionable [17, 47]. SRH is a wide-ranging subject consisting of a mixture of sensitive but important issues; therefore, reliable knowledge is essential for improving quality of life and minimising the risk of disease or harmful practices [48, 49]. In this context, parents, teachers and doctors can be the preferred sources of information for adolescents. Unfortunately, their communication with adolescents about SRH issues is rare and begins in the older age group, as is evident from earlier qualitative studies carried out in Ethiopia and Uganda, where it was believed that adolescents’ awareness of SRH would make them sexually active [50, 51]. Adolescents, like other members of society, are entitled to basic universal rights; nonetheless, they have limited ability to exercise these rights, as featured in our research and also in research work done in Bangladesh and Zimbabwe [52, 53].

Overall, this research reveals a gap in perceptions about ASRHR in Lahore (Punjab), due to various influencing determinants. In our study, parental education and household wealth have been consistently statistically significant, which is comparable to studies conducted in Ghana and Uganda [54, 55]. This research also highlights a relatively low perception amongst parents due to lack of knowledge and cultural values, demonstrating the ‘denial of rights’ in the SRH arena for adolescents, as is apparent in studies from Ethiopia and Kenya [56–58]. Contrarily to the previous researches, this study shows that perception of ASRHR is higher amongst younger and uneducated adolescents [55, 56, 59]. Nevertheless it reveals no significant relationship with adolescents’ age and education. This indicates their curious nature with spending more time on electronic media to explore, however, it also questions the correct and complete knowledge regarding SRHR [48, 60]. These findings also draw attention to the fact that younger age and low level of education or uneducated status of adolescents predisposes them to adopt negative behaviour, which further aggravate the various types of health risks and social problems, if not guided properly [60, 61].

Multiple socio-cultural and structural barriers hindering adolescents’ access to requisite information and autonomy is also confirmed by other research. These include, but are not limited to traditional cultural norms, lack of education and open discussion, a communication gap between parents/teachers and adolescents, and a non-supportive environment amongst key stakeholders [32, 62, 63]. Here, the role of parents, teachers and doctors is essential to provide an enabling environment for adolescents and to give them confidence to share their SRH problems, irrespective of any hesitation. Thus, community-based and peer education programmes are required to equip parents, teachers and peers with practical SRH knowledge to promote health behaviours focusing on ASRHR.

Notwithstanding, there were certain limitations encountered by this research due to the nature of the topic and taboos associated with discussion on SRH. Key limitations faced included hesitation and non-cooperative behaviour of respondents particularly of adolescents under 18 years of age, which resulted into refusal to participate or withdraw from research after providing few responses (in the form of skipped questions). This hesitation of discussion due to sensitive approach towards the topic was more common in rural areas, where field teams had to approach more households for an interview as compared to urban areas. The refusal to participate may bias the results, because the experiences of specific groups may be underrepresented.

## Conclusion

The research revealed a low level of perception amongst adolescents and their parents/caregivers on ASRHR in the district of Lahore emphasising a rights-based approach. The research demands to design specific policies and educational programmes to address the communication gap and basic educational needs to promote healthy practices among communities. Summarising the findings, the research recommends advocacy of the Punjab Government’s departments such as Youth Affairs, Education, Health, Human Rights and Minority Affairs, Information and Culture, and Literacy and Non Formal Basic Education as well as community members including parents, teachers, doctors, religious scholars and media groups to empower adolescents with health education. This can be done through use of local newspapers and cable networks, the inclusion of SRH-related topics in educational curricula, establishing virtual knowledge centres, encouraging debate competitions, organising orientation sessions for professionals/experts and local community meetings etc. This would be helpful in boosting adolescents’ confidence and personal development, ensuring that they will not be deceived, maltreated, or exploited by anybody else.

## Abbreviations

ASRHR: Adolescents sexual and reproductive health rights
FGD: Focus group discussion
ICPD: International Conference on Population and Development
IDI: In-depth interviews
SRH: Sexual and reproductive health
SRHR: Sexual and reproductive health rights
